# Report of a Rare Case of Intracranial Extramedullary Hematopoiesis Mimicking a Brain Tumor

**DOI:** 10.7759/cureus.37491

**Published:** 2023-04-12

**Authors:** Tehmina Habib, Mohammad Abu-Abaa, Emily Chen

**Affiliations:** 1 Internal Medicine Residency Program, Capital Health Regional Medical Center, Trenton, USA

**Keywords:** myelofibrosis, essential thrombocythemia, headache, intracranial mass, extra-medullary hematopeisis

## Abstract

Failure of the bone marrow to maintain adequate blood cell production to match blood metabolic demand incites the production of cell lines outside the bone marrow, which is known as extramedullary hematopoiesis. Herein, we are reporting an 80-year-old male patient who presented with two weeks of worsening headaches and behavioral changes. Labs showed thrombocytosis and imaging showed a large right-sided hemorrhagic brain mass. No evidence of malignancy was seen elsewhere. Brain mass biopsy showed intracranial extramedullary hematopoiesis (IEMH) and bone marrow biopsy confirmed the diagnosis of essential thrombocythemia (ET)/myelofibrosis. This case adds to a few reported cases of IEMH, and to the best of our knowledge, this is the first reported case of IEMH in association with ET. It helps remind clinicians to keep IEMH in the differential diagnosis of those presenting with signs and symptoms of elevated intracranial pressure (ICP) and a newly found brain mass on the background of previously diagnosed or suspected myeloproliferative neoplasms.

## Introduction

Essential thrombocythemia (ET) is an uncommon clonal myeloproliferative neoplasm characterized by increased platelet count and platelet dysfunction [1]. Like other myeloproliferative neoplasms, it can progress to myelofibrosis and can be associated with extramedullary hematopoiesis (EMH). EMH is a tumor-like proliferation of hematopoietic tissue secondary to chronic anemia. This usually happens in the liver, spleen, kidneys, and lymph nodes, but also has been reported in the thymus, adrenal glands, ovary, and pelvic region [2]. The occurrence of intracranial EMH is extremely rare and can involve the dura, falx, optic nerve sheath, or brain parenchyma [3].

## Case presentation

**Figure 1:**
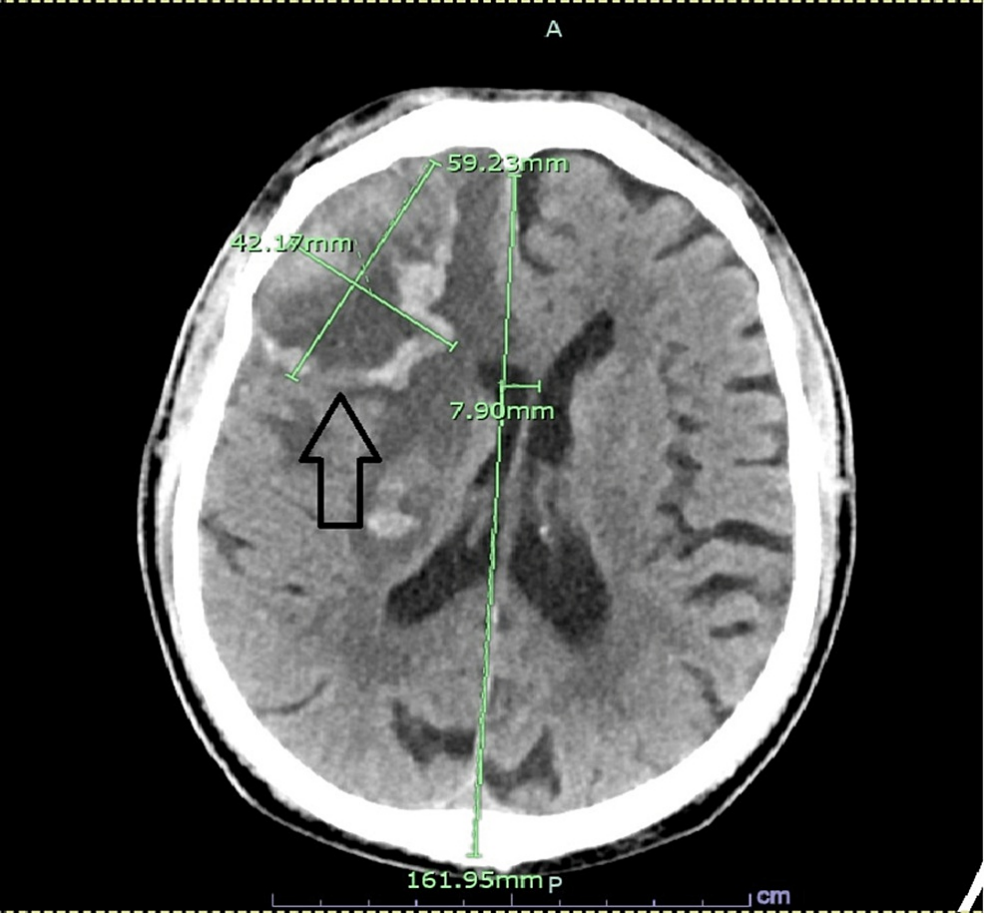
Cross-Sectional Computed Tomography (CT) Scan Head CT scan showing an irregularly shaped hemorrhagic mass in the right anterior frontal lobe with an 8 mm midline shift to the left (arrow)

**Figure 2:**
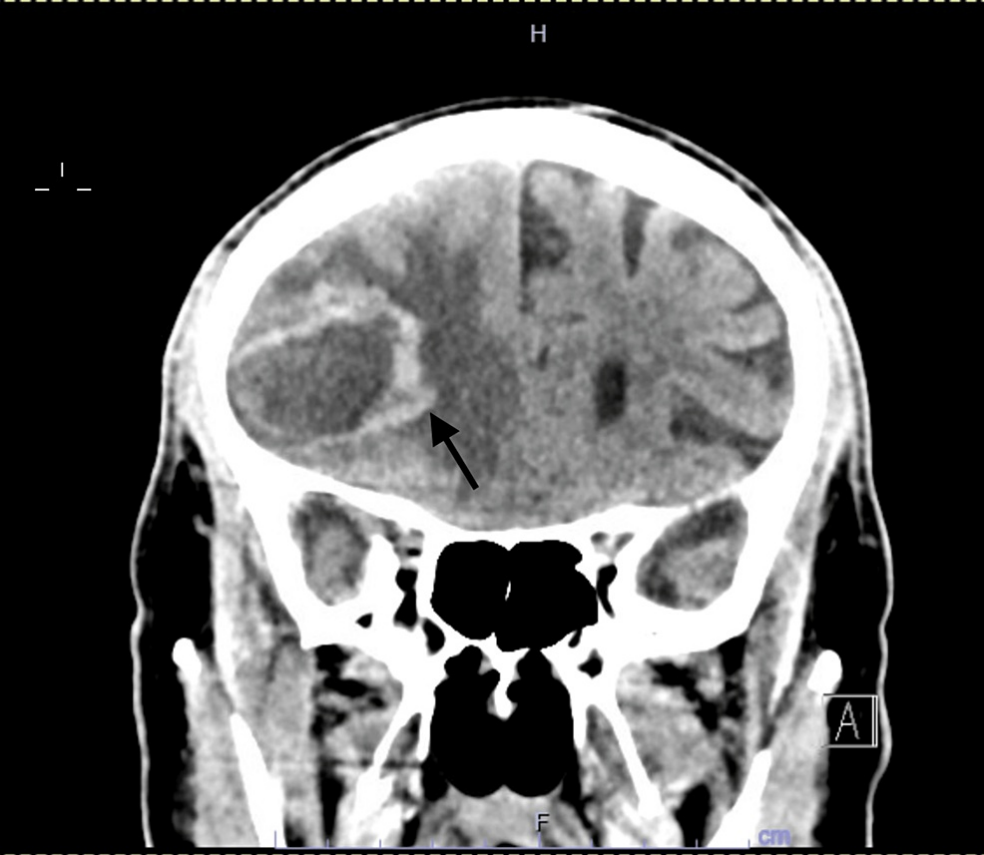
Coronal CT Head Coronal CT head showing the heterogenous right-sided mass (arrow)

An 80-year-old male patient presented to the emergency department (ED) as a referral from his primary care physician for an abnormal computed tomography (CT) head scan result. The patient was complaining of two weeks of diffuse constant headaches and behavioral changes. It was described that during these two weeks, he had progressive forgetfulness, sleepiness, and somnolence. On multiple occasions, he forgot his way home. No preceding history of recent trauma, fall, or infection was reported. Past medical history was significant for ischemic stroke on aspirin, diabetes type 2, and hypertension. CT scan showed a large, 5.9 x 4.2 x 3.7 cm, peripherally hyperattenuating and internally hypoattenuating irregularly shaped heterogeneous hemorrhagic mass in the right anterior frontal lobe with extensive surrounding vasogenic edema, mass effect on the right lateral ventricle, and 8 mm midline shift to the left. It also showed a separate 1.5 x1.0 x 1.4 cm hemorrhagic lesion in the right thalamus involving the internal capsule with surrounding vasogenic edema (Figures 1, 2). In the ED, vital signs included a temperature of 36.8 degrees Celsius, heart rate of 56 beats per minute, respiratory rate of 18 cycles per minute, blood pressure of 160/60 mmHg, and oxygen saturation (SpO2) of 97%. On physical exam, he was alert and fully oriented, had clear speech, bilateral reactive and equal pupils at 3 mm, intact visual field bilateral, subtle left-sided facial droop, subtle left-sided tongue deviation, Medical Research Council (MRC) muscle power 5/5 on all four extremities with a pronator drift on the left lower extremity, and diminished light touch sensation over the right upper and lower extremities. Otherwise, the physical exam was unremarkable. Basic labs showed macrocytic anemia with hemoglobin of 11.9 g/dl and a mean corpuscular volume (MCV) of 95 fL (reference 79-95 fL) and mild leukocytosis 10,500 cells/ml (reference 4000-10,100). It also showed thrombocytosis of 937,000 cells/ml along with hyperkalemia of 5.6 mmol/L and normal renal function; see Table 1 for complete lab trends. A clevidipine infusion was started, aspirin was held, and he was admitted to the neurocritical care unit.

**Table 1:** Labs Trends Lab finding trends throughout the period of hospital stay

Variable	On Admission	Day 3 Hospitalization	On Discharge	Reference Range
WBC	10,500	13,750	13,250	4,000-10,100 cells/ml
Hemoglobin	11.9	10.9	9.5	13.7-17.5 g/dl
MCV	95	94	97	79-95 fL
Platelets	937,000	1,044,000	722,000	150-400 cells/mcl
Sodium	137	136	138	137-145 mmol/L
Potassium	5.6	4.8	4.4	3.5-5.1 mmol/L
Calcium	9.8	9.2	8.9	8.6-10.3 mg/dl
Creatinine	1.09	1.23	1.30	0.66-1.25 mg/dl
AST	35	-	-	17-59 U/L
ALT	25	-	-	0-49 U/L
Total Bilirubin	0.7	-	-	0.2-1.3 mg/dl

**Figure 3:**
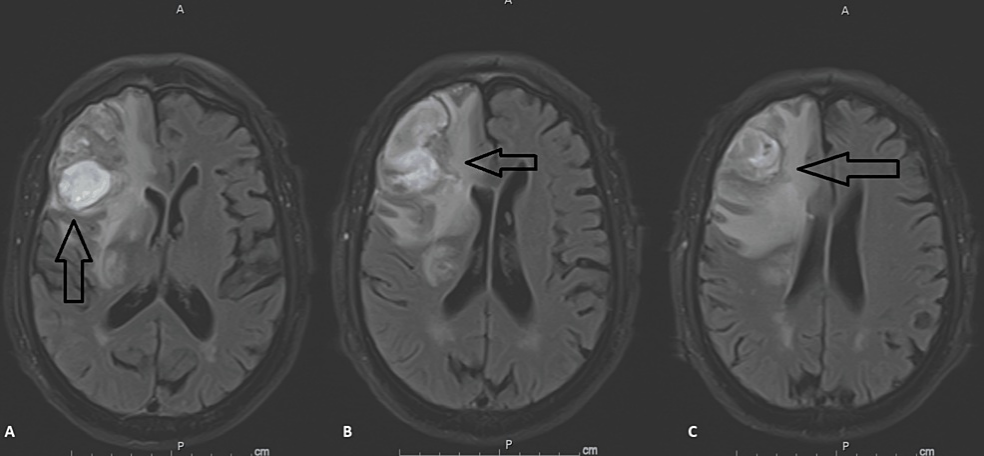
Magnetic Resonance Imaging (MRI) Brain MRI fluid-attenuated inversion recovery (FLAIR) brain showing enhancing irregular heterogeneous right frontal mass (arrows in A, B, and C)

**Figure 4:**
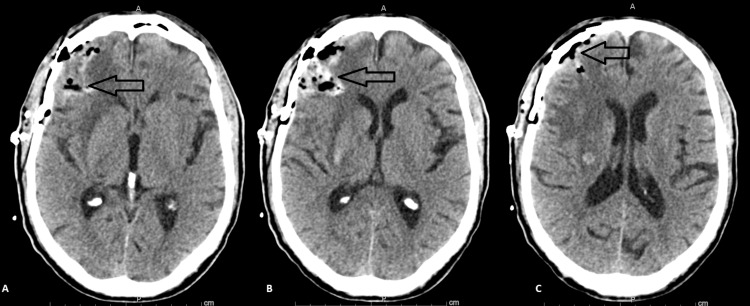
Post-Craniotomy CT Head Post-craniotomy CT head with a residual defect after resection (arrows in A, B, and C)

**Figure 5:**
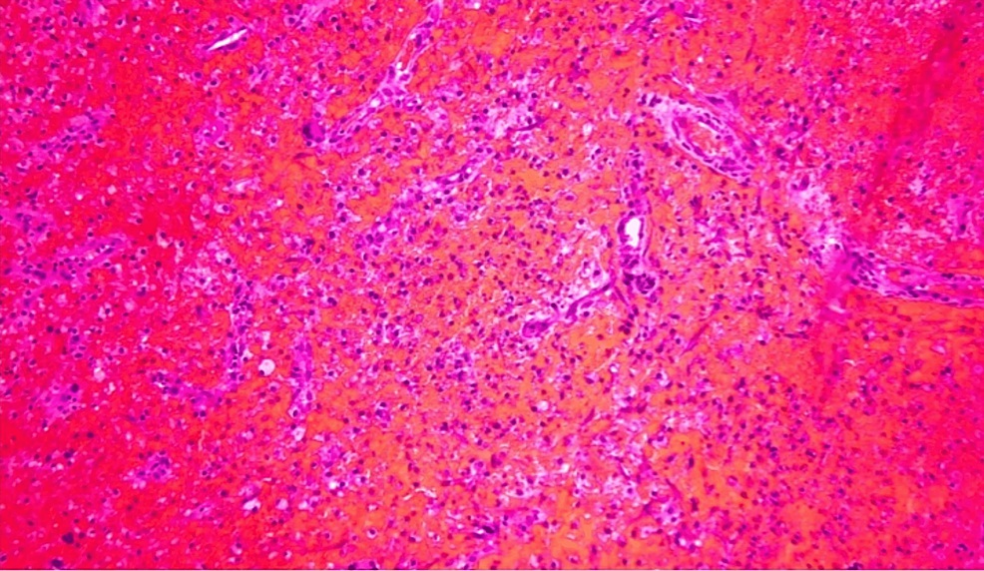
Brain Mass Biopsy Brain mass biopsy histological examination showing atypical megakaryocytes

CT scan screening for cancer, including the abdomen, pelvis, and chest, were all unremarkable for organomegaly or any mass lesion. No cardiac abnormality was seen on transthoracic echocardiography and cardiac rhythm was sinus. Brain magnetic resonance imaging (MRI) showed stable, similar findings to the CT scan with superimposed enhancement as well as a leptomeningeal enhancement (Figure 3). CT angiography brain and neck were also unremarkable for aneurysm or arteriovenous malformation and the hemorrhagic masses remained stable on repeat CT/MRI four days later. Right-sided craniotomy for resection of hemorrhagic brain mass was pursued and a CT scan head was obtained postoperatively (Figure 4). Pathological examination of the obtained brain specimen showed extensive acute and organizing hemorrhages with foci of atypical cells, some of which with mitotic activity. CD-45 highlighted many inflammatory cells. CD-10 indicated extensive immunoreactivity with abundant CD-68 positive macrophages and increased KI-67. These cells were negative for CK7, CK20, MART1, S100, Napsin A, CDX2, PSA, AE1/AE3, and HMB45 but positive for CD-61 (Figure 5). These were suggestive of atypical megakaryocytes and clusters of nucleated erythroid precursors indicative of extramedullary hematopoiesis. Peripheral blood showed moderate anisopoikilocytosis, normally granulated and lobulated granulocytes, increased number of platelets with giant platelets, and left-shifted granulocytic maturation. Platelet count continued to increase up to 1,044,000 cells/ml. Serum folate and B12 were within normal limits. Erythropoietin level was also within normal limits at 15.4 ml international unit/ml (reference is 2.6-18.5). JAK 2 V617F mutation (VAF 25%) was also positive. Both aspirin and hydroxyurea were started. Bone marrow biopsy showed atypical megakaryocytes with focal clustering with no increase in CD-34 and CD-117 blasts and no ring sideroblasts. Reticulin stain showed grade MF-1 fibrosis. Cytogenetic analysis was unremarkable. The patient was diagnosed with a myeloproliferative neoplasm (MPN) of non-CML type. The patient remained stable neurologically allowing for safe discharge home.

## Discussion

Essential thrombocythemia (ET) is an uncommon myeloproliferative disorder with an average age of diagnosis of 58 years [1]. ET has a high risk of progression to myelofibrosis or acute leukemia. ET is an infrequent cause of thrombocythemia accounting for less than 2% of cases with an incidence of 1.2-3 per 100,000 annually. The most common mutation in ERT is JAK V617F in 60% followed by CALR in 20% and MPL in 3% [1]. Diagnosis of ET is based on thrombocythemia, bone marrow biopsy, and genetic testing to help differentiate ET from other myeloproliferative disorders as well as etiologies of reactive thrombocytosis.

Extramedullary hematopoiesis (EMH) is a process through which the body compensates for long-standing anemia secondary to poor blood cell production by the bone marrow or hemolysis by the proliferation of pluripotent stem cells outside the bone marrow. It is usually seen in hemoglobinopathies, including sickle cell anemia, thalassemia, and hereditary spherocytosis, and myeloproliferative disorders, including myelofibrosis, polycythemia rubra vera, and myeloid metaplasia [2]. However, it has also been reported in other etiologies of ineffective erythropoiesis such as HIV-induced bone marrow suppression [4]. This was also reported in association with multiple myeloma [5]. There are two types including para-osseous and extra-osseous EMH. Para-osseous EMH occurs as a result of the rupture of medullary tissue to surrounding soft tissue and is usually seen in hemoglobinopathies. On the other hand, the extra-osseous type occurs as a result of the proliferation of pluripotent stem cells in soft tissues [6].

Although paraspinal EMH is a well-known condition. There are only a few reported cases of intracranial EMH (IEMH). Current knowledge is based on isolated case reports and hence there is no known epidemiological data. IEMH has been reported to arise from the cranial base close to the sphenoid or ethmoid bones and thus presents initially with progressive visual deterioration [7]. IEMH originating from the choroid plexus and presenting mainly with signs and symptoms of increased ICP was also reported [8]. Intraparenchymal lesions have also been reported [9].

Intracranial EMH might be explained by the mesenchymal origin of pluripotent stem cells in the central nervous system (CNS) [10]. In addition, the fact that CNS cells are capable of producing hematopoietic growth factors that can contribute to EMH might explain it [11]. However, most of the reported cases of IEMH did not show evidence of EMH outside the central nervous system (CNS), similar to our case. No explanation was found in the literature review regarding this finding.

Similar to our case, IEMH can be the initial presentation of myeloproliferative disorder or hemoglobinopathy, although most of the reported cases had an established diagnosis of hemoglobinopathy or myeloproliferative disorder prior to the diagnosis of IEMH [9]. Although patients might remain initially clinically silent, signs and symptoms of elevated intracranial pressure (ICP) eventually develop, including headache, visual disturbance, papilledema, and motor and sensory deficits. The diagnosis is first suggested by computed tomography (CT) finding of single/multiple extradural masses with attenuation equal to or greater than that of the gray matter with contrast enhancement [6]. Magnetic resonance imaging (MRI) shows the characteristic hyperintensity on T2, diffusion-weighted imaging (DWI) attenuated diffusion coefficient (ADC) sequences, which is explained by the magnetic susceptibility of hemosiderin [12].

Multiple cases were reported in the literature citing IEMH, see Table 2. Except for one case, IEMH was not the initial presentation, in contrast to our case. Those patients were already diagnosed with underlying ineffective hematopoiesis and presentation was mainly secondary to elevated intracranial pressure (ICP). Focal neurological deficits and seizures were also reported in the initial presentation. To the best of our knowledge, there is no reported case of essential thrombocythemia in association with IEMH, making this reported case the first in the medical literature. Similar to our patient, there was no evidence of EMH in the liver or spleen at the time of diagnosis of IEMH in most of the reported cases.

**Table 2:** Reported Cases of IEMH Reported cases of IEMH in the literature with their respective characteristics IEMH: intracranial extramedullary hematopoiesis

Reference	Age in Years	Initial Presentation With IEMH	Underlying Pathology	Site of IEMH	Clinical Presentation	Associated Sites of EMH
O. Ayyildiz et al. [13]	18	No	Primary Myelofibrosis	Meningeal	Headache	Hepatosplenomegaly
D. Morrar et al. [9]	67	Yes	Idiopathic	Intraparenchymal	Headache	None
H. Tabesh et al. [8]	34	No	Beta Thalassemia	Choroid Plexus	Headache and visual loss	Hepatomegaly
Jose-Alberto Palma et al. [5]	77	No	Multiple Myeloma	Extra-axial	Disorientation and obtundation	None
BL. Koch et al. [10]	69	No	Primary Myelofibrosis	Extra-axial	Hemiparesis	None
B. Karki et al. [14]	13	No	Beta Thalassemia	Extra-axial	Hemiparesis	None
A. Singer et al. [15]	54	No	Primary Myelofibrosis	Extra-axial	Headache	None
S. Fucharoen et al. [16]	23	No	Beta Thalassemia	Extra-axial	Seizure	None
A. Musolino et al. [4]	36	No	HIV	Meningeal	Headache	None
S. Al-Umran et al. [17]	26	No	Sickle Cell Disease	Meningeal	Seizure	None
J. Boban et al. [18]	48	No	Polycythemia Rubra Vera	Intraparenchymal	Tinnitus	Splenomegaly

## Conclusions

IEMH is an extremely rare entity. It is usually seen in association with chronic hemoglobinopathies and myeloproliferative disorders. Initial presentation is usually with signs and symptoms of elevated ICP. It can be seen in intraparenchymal, meningeal, extra-axial, or choroid plexus locations. This is the first reported case of ET in association with IEMH.
